# Association between gut microbiota and malignant cardiac tumors: A two‐sample Mendelian randomization study

**DOI:** 10.1002/cam4.7455

**Published:** 2024-07-02

**Authors:** Yongfei Song, Jiale Hu, Chongrong Li, Jiangfang Lian

**Affiliations:** ^1^ Ningbo Institute of Innovation for Combined Medicine and Engineering Ningbo Medical Center Lihuili Hospital, Ningbo University Zhejiang China; ^2^ Department of Cardiology Ningbo Medical Center Lihuili Hospital, Ningbo University Zhejiang China

**Keywords:** cardiac tumor, gut microbiota, Mendelian randomization, SNPs

## Abstract

**Background:**

Recent studies provide compelling evidence linking the gut microbiota to most cancers. Nevertheless, further research is required to establish a definitive causal relationship between the gut microbiota and malignant cardiac tumors.

**Methods:**

The genome‐wide association studies (GWAS) data on the human gut Microbiota, included in the IEU Open GWAS project, was initially collected by the MiBioGen consortium. It encompasses 14,306 individuals and comprises a total of 5,665,279 SNPs. Similarly, the GWAS data on malignant cardiac tumors, also sourced from the IEU Open GWAS project, was initially stored in the finnGen database, including 16,380,303 SNPs observed within a cohort of 174,108 individuals within the European population. Utilizing a two‐sample Mendelian randomization (MR) methodology, we examined whether there exists a causal association between the gut microbiota and cardiac tumors. Additionally, to bolster the credibility and robustness of the identified causal relationships, we conducted an extensive array of sensitivity analyses, encompassing Cochran's *Q* test, MR‐PRESSO tests, MR‐Egger interpret test, directionality test and leave‐one‐out analysis.

**Results:**

Our analysis unveiled seven distinct causal associations between genetic susceptibility in the gut microbiota and the incidence of malignant cardiac tumors. Among these, the Family Rikenellaceae, genus *Eubacterium* brachy group, and genus *Ruminococcaceae* UCG009 exhibited an elevated risk of cardiac tumors, while the phylum Verrucomicrobia, genus *Lactobacillus*, genus *Ruminiclostridium*5, and an unknown genus id.1868 were genetically linked to a reduced risk of cardiac tumors. The causal relationship between these two bacteria, belonging to the phylum Verrucomicrobia (OR = 0.178, 95% CI: 0.052–0.614, *p* = 0.006) and the genus *Ruminococcaceae* UCG009 (OR = 3.071, 95% CI: 1.236–7.627, *p* = 0.016), and cardiac tumors was further validated through sensitivity analyses, reinforcing the robustness and reliability of the observed associations.

**Conclusion:**

Our MR analysis confirms that the phylum Verrucomicrobia displays significant protection against cardiac tumor, and the genus *Ruminococcaceae* UCG009 leads to an increasing risk of cardiac tumor.

## 1 INTRODUCTION

Cardiac tumors are considered rare, with a reported incidence ranging from 1.38 to 30 cases per 100,000 individuals annually. Among primary cardiac tumors, approximately 20% are of malignant nature.^1^, ^2^ Clinical manifestations of malignant cardiac tumors exhibit considerable heterogeneity. While some individuals may remain asymptomatic, the detection of tumors occurs fortuitously during imaging examinations conducted for unrelated reasons.^3^, ^4^ Nevertheless, symptomatic cases can be classified into constitutional symptoms characterized by fever and weight loss, distal embolization, or direct effects resulting from the presence of the malignant tumor. Distal embolization refers to the detachment of tumor fragments or formation of blood clots, which subsequently traverse the circulatory system and lodge in distant organs, contributing to the development of various clinical conditions such as stroke, mesenteric ischemia, renal infarction, or acute limb ischemia.^5^ Obstruction, cardiac tamponade, and arrhythmias represent the direct consequences of malignant cardiac tumors.^6^ Obstructive symptoms encompass congestive heart failure or syncope, arising from compromised blood flow within the cardiac chambers or valves. Cardiac tamponade, the most severe and potentially life‐threatening presentation, emerges when fluid accumulates within the pericardial space, impeding adequate cardiac filling. Disruption of normal myocardial function due to malignant cardiac tumors can lead to the manifestation of diverse arrhythmias, including atrial fibrillation, ventricular tachycardia, or ventricular fibrillation.^7^, ^8^ Given the low incidence of cardiac tumors and the limited reports on this subject over the past decade, there is a paucity of studies exploring the molecular mechanisms underlying these tumors.^9^ Therefore, it is crucial to investigate and elucidate the molecular basis of malignant cardiac tumors to enhance our understanding and promote the development of preventive and therapeutic strategies.

The gut microbiota has gained significant recognition as a crucial modulator of human health, exerting its influence not only within the gastrointestinal tract but also extending to distal organs such as the brain, liver, and pancreas.^10^ Perturbations in the composition and functionality of the gut microbiome, termed dysbiosis, have been associated with the development of various pathological conditions, such as obesity, diabetes, and neurodegenerative diseases.^11^ Furthermore, emerging evidence suggests a potential link between bacterial infection and cancer initiation.^12^ A prominent example is the colonization by *Helicobacter pylori*, which causes persistent inflammation and gastritis, potentially leading to gastric malignancy in some infected individuals. Conversely, the eradication of *H*. *pylori* has been shown to reduce the risk of gastric cancer, underscoring its involvement in the early stages of gastric carcinogenesis.^13^, ^14^ Additionally, investigations into colorectal cancer (CRC) have identified specific bacteria enriched in the fecal metagenomic samples of CRC patients, such as *Bacteroides fragilis* and *Thermanaerovibrio acidaminovorans*.^15^ These bacterial markers could potentially serve as diagnostic tools across diverse populations. However, despite advancements in our understanding of the impact of gut microbiota on various tumors, its role in cardiac tumors remains uncertain. There has been limited research into how gut microbiota dysbiosis might affect the development and progression of cardiac tumors, particularly malignant ones. Unraveling this relationship could lead to the discovery of new therapeutic targets and diagnostic markers for malignant cardiac tumors, thereby potentially improving patient outcomes in this relatively understudied field.

Mendelian randomization (MR) is a robust method used for estimating causal relationships between exposures and outcomes by leveraging genetic variation in nonexperimental data.^16^, ^17^ This approach has found widespread application in causal inference studies investigating various types of tumors and the gut microbiota. Notably, Zixin Wei revealed that a higher abundance of Sellimonas is associated with an increased risk of estrogen receptor‐positive breast cancer, while a higher abundance of *Alphaproteobacteria* seem to inhibit the development of prostate cancer.^18^ Additionally, Jun Ma confirmed a correlation between the presence of *Ruminococcaceae*, *Porphyromonadaceae*, *Bacteroidetes*, and a decreased risk of liver cancer.^19^ In our study, we utilized five MR methods to evaluate the causal associations between gut microbiota and malignant neoplasms of the heart, mediastinum, and pleura. Subsequently, we conducted a series of sensitivity analyses to ensure the robustness of our findings. Ultimately, our results identified two distinct groups of gut microbiota, namely the genus *Ruminococcaceae* UCG009 and the phylum Verrucomicrobia, each showing positive and negative causal relationships, respectively, with cardiac tumors. These findings contribute to the growing body of knowledge on the potential role of gut microbiota in the development of cardiac tumors and highlight the importance of further research in this area.

## 2 MATERIALS AND METHODS

### 2.1 Study design

The primary goal of this study is to investigate 211 specific gut microbiota taxa, with a particular focus on their relationship with malignant tumors in the heart, mediastinum, and pleura. To achieve this, a preliminary screening is conducted to identify gut microbiota communities that exhibit a significant association with these specific tumor types. Subsequently, a detailed Mendelian randomization (MR) analysis is performed to gain deeper insights into the observed relationships.

The MR analysis is guided by three underlying assumptions. First, it assumes that the instrumental variables (IVs) utilized in the analysis are indeed associated with the exposure factor being studied. Second, the independence of the IVs from any potential confounding factors is assumed, ensuring that the identified associations are not influenced by extraneous variables. Finally, the analysis assumes that the effects of the IVs on outcomes are solely mediated through the exposure factor, thus providing a clearer understanding of the underlying mechanisms at play.

### 2.2 Data acquirement

Data on single nucleotide polymorphisms (SNPs) related to the gut microbiota was sourced from the latest meta‐analysis of genome‐wide association studies (GWAS) provided by the MiBioGen consortium. This comprehensive analysis incorporated data from 18 cohorts, encompassing a total of 14,306 individuals.^20^ The study aimed to evaluate the presence and composition of 211 taxonomic levels, including kingdom, phylum, class, order, family, genus, and species,^21^ within the gut microbiota. Three distinct variable regions in the bacterial genome, including V4, V3‐V4, and V1‐V2, were used to assess the gut microbiota.

The GWAS dataset, labeled “finn‐b‐C3_HEART_MEDIASTINUM_PLEURA_EXALLC” in IEU Open GWAS project, utilized as the outcome in our study, originates from the Finngen Biobank, which includes genetic data from 174,108 individuals (102 case and 174,006 control) as of 2021.^22^ This dataset provides valuable genetic insights into malignant tumors of the heart, mediastinum, and pleura, serving as a crucial resource for exploring genetic associations with these rare cancer types.

It is important to note that the original studies utilizing these data sources have already received ethical approval from the respective review committees. Therefore, the current study does not require additional ethical clearance, as it builds upon preexisting approved research initiatives. By leveraging these comprehensive datasets, this study endeavors to shed light on the complex interplay between genetic factors, gut microbiota composition, and the occurrence of malignant tumors affecting the heart, mediastinum, and pleura.

### 2.3 Genetic instrument selection

The association between instrumental variables (IVs) and gut microbiota was evaluated using a stringent threshold of *p* < 10E−5. The selection process encompassed the following specific steps, guided by established methodologies.^23^

(1) The TwoSampleMR package in R software was employed to extract relevant single nucleotide polymorphisms (SNPs) from the summarized data of gut microbiota genome‐wide association studies (GWAS). This allowed for the identification of SNPs associated with the exposure of interest.

(2) To address linkage disequilibrium and minimize confounding effects, SNPs were grouped using a clustering approach if they exhibited pairwise correlation (*r* ^2^) exceeding 0.001 within a genomic distance of 10,000 base pairs (kb).

(3) To ensure data integrity, any SNPs displaying overlapping or echoing sequences were systematically excluded from the analysis. This step aimed to eliminate potential biases arising from duplicated or misaligned genomic regions.

(4) The remaining SNPs were cross‐referenced with GWAS data pertaining to cardiac tumor. This comparison facilitated the identification of any shared genetic variants between the gut microbiota and cardiac tumor, serving as an important control for confounding factors that may influence the observed IV‐exposure association (referred to as MR hypothesis II).

(5) To further scrutinize potential confounding effects, a comprehensive phenotype scanner (available at http://www.phenoscanner.medschl.cam.ac.uk/) was utilized.^24^ This tool enabled a thorough exploration of all SNPs displaying positive associations with relevant phenotypes, facilitating the identification of any additional confounding variables that could impact the IV‐exposure relationship.

(6) The robustness of the instrumental variables was evaluated utilizing the F‐statistic, a commonly employed measure in MR analyses. Specifically, an *F*‐statistic threshold of *F* < 10 was employed to identify weak instrument bias. The *F*‐statistic is calculated as *F* = [(*n* − *k* − 1) / *k*] * [*R* ^2^ / (1 − *R* ^2^)], where *n* represents the sample size and *k* denotes the number of IVs. *R* ^2^ quantifies the proportion of exposure variation explained by genetic variation, estimated as 2 * EAF * (1 − EAF) * beta^2^, with EAF denoting the effect allele frequency and beta2 representing the effect size. The *F*‐statistic serves as a quantitative measure of the magnitude of the association between the instrumental variables (IVs) and the specific exposure being investigated.

### 2.4 Statistical analysis

Our primary analytical approach involved using the Inverse Variance Weighted (IVW) method, supplemented by the weighted median, MR‐Egger, Weighted mode, and Simple mode tests as additional analysis techniques. To address potential outlier effects, we also conducted robust analyses using the IVW and MR‐Egger methods. The MR‐Egger intercept test and Outlier (MR‐PRESSO) analysis were utilized to evaluate the presence of horizontal pleiotropy.^25^, ^26^ Heterogeneity was assessed by calculating Cochran's *Q* statistic. The rationale behind our selection of statistical tests is as follows: The Instrumental Variable Weighted (IVW) method assumes the validity of all single nucleotide polymorphisms (SNPs) utilized as instrumental variables and estimates the overall effect as a weighted average based on the inverse‐variance of the instrumental variable effects. IVW demonstrates statistical robustness in the absence of horizontal pleiotropy.^27^ To account for directional pleiotropy, a significant deviation from a zero intercept in the MR‐Egger intercept test indicates the presence of significant horizontal pleiotropy. The MR‐PRESSO approach assesses the magnitude of horizontal pleiotropy by summarizing the residuals for each SNP. By adjusting for horizontal pleiotropy, the primary IVW analysis results can be obtained. The MR‐PRESSO global test evaluates the overall level of horizontal pleiotropy, while the outlier test identifies outlier SNPs responsible for the observed horizontal pleiotropy.^26^ Additionally, a leave‐one‐out analysis was conducted, where the effects of each individual SNP were calculated after excluding it from the analysis. Inverse MR analysis was performed to determine the direction of causal relationships. The Steiger test, employed within the MR‐Pleiotropy Test (MRPRESSO) framework, was utilized to assess the causal directionality between gut microbiota and cardiac tumor. A *p*‐value of less than 0.05 would indicate that the identified exposure significantly contributes to the outcome, affirming the hypothesized causal relationship.

The statistical analyses were performed using R software version 4.1.1. Several R packages were employed, including “TwoSampleMR” Version 0.5.6, “MendelianRandomization” Version 0.9.0,^28^ and “MRPRESSO” Version 1.0.

## 3 RESULTS

### 3.1 Screening IVs and removal confounding factors

Initially, we selected 2637 SNPs as instrumental variables (IVs) for the 211 gut microbiota groups. Based on the IVW *p*‐values with a threshold of *p* < 0.05, we identified seven gut microbiota communities. To account for the potential influence of confounding factors, we conducted a query using Phenoscanner for the SNPs associated with the positive results mentioned above. No SNPs related to the confounding factors were found (Data S1). The results of all initial MR analyses are presented in Data S2.

### 3.2 Assessment of causal relationship and sensitivity analysis

**FIGURE 1:**
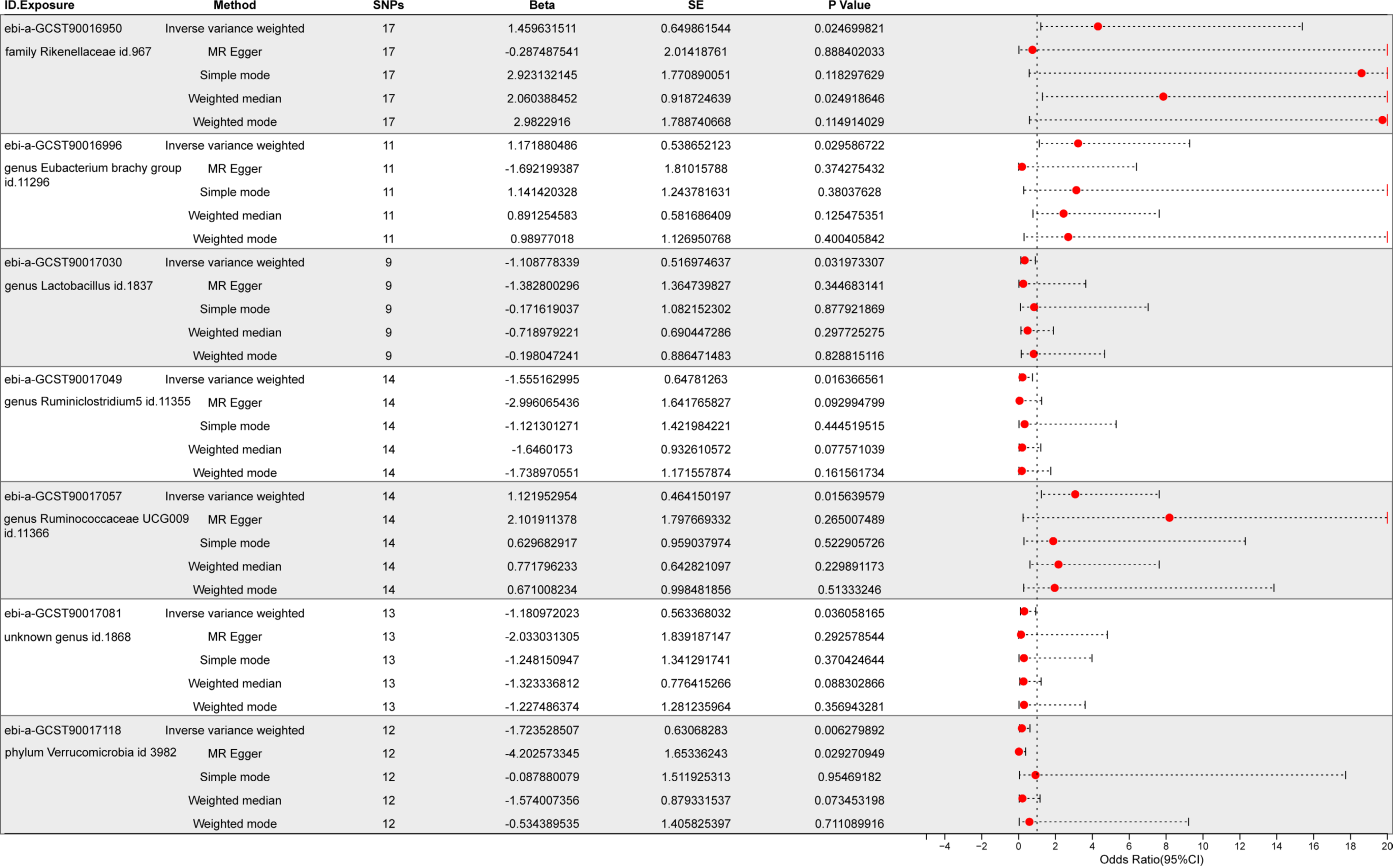
Potential gut microbiota associated with cardiac tumor. This forest plot shows the correlation between gut microbiota and cardiac tumor risk. The horizontal axis represents the odds ratio (OR). A central line is set at OR = 1, indicating no significant association. Significance is denoted by *p* < 0.05.

Given the stronger statistical power of IVW, our results primarily rely on the IVW method. As shown in the forest plot (Figure 1), the IVW analysis revealed that family Rikenellaceae id.967 (OR = 4.304, 95% CI: 1.204–15.384, *p* = 0.025), genus *Eubacterium* brachy group id.11296 (OR = 3.228, 95% CI: 1.123–9.278, *p* = 0.03), genus *Ruminococcaceae* UCG009 id.11366 (OR = 3.071, 95% CI: 1.236–7.627, *p* = 0.016) were associated with an increased risk of heart tumors, while phylum Verrucomicrobia id.3982 (OR = 0.178, 95% CI: 0.052–0.614, *p* = 0.006), genus *Lactobacillus* id.1837 (OR = 0.33, 95% CI: 0.12–0.909, *p* = 0.032), genus *Ruminiclostridium*5 id.11355 (OR = 0.211, 95% CI: 0.059–0.752, *p* = 0.016), and unknown genus id.1868 (OR = 0.307, 95% CI: 0.102–0.926, *p* = 0.036) were associated with a decreased risk of heart tumors (Figure 1). To elucidate the causal direction between gut microbiota and cardiac tumors, we utilized the Steiger test. Our results revealed that the seven specified types of gut microbiota are implicated in the development of cardiac tumors, a conclusion that is markedly significant with a *p*‐value less than 0.05 (Table 1).

**TABLE 1:** Heterogeneity test and pleiotropy test for MR results.

	*Q*	Egger_interpreter	Egger_interpreter_*p*val	Steiger_*p*val	MRPRESSO_global
ebi‐a‐GCST90016950	0.537	0.131	0.374	4.524E‐73	0.524
ebi‐a‐GCST90016996	0.073	0.385	0.134	4.468E‐41	0.090
ebi‐a‐GCST90017030	0.837	0.035	0.834	5.616E‐46	0.859
ebi‐a‐GCST90017049	0.740	0.111	0.358	2.559E‐61	0.752
ebi‐a‐GCST90017057	0.932	−0.104	0.583	1.690E‐58	0.942
ebi‐a‐GCST90017118	0.619	0.237	0.136	1.091E‐49	0.677
ebi‐a‐GCST90017081	0.408	0.084	0.635	1.475E‐54	0.420

**FIGURE 2:**
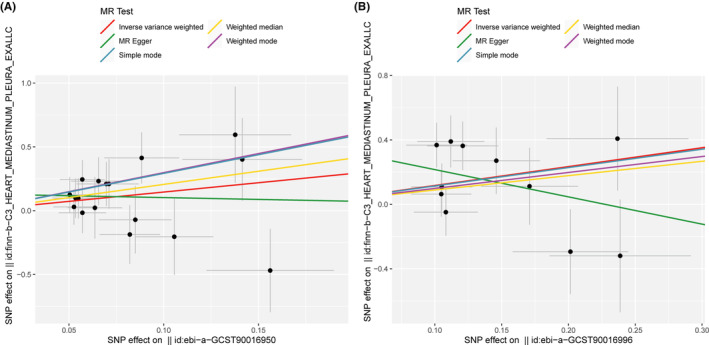
Excluding two gut microbiota that IVs with opposite effect in different Mendelian randomization (MR) analysis models. Horizontal ordinate represents the effects of SNPs on family Rikenellaceae id.967 and genus *Eubacterium* brachy group id.11296, respectively; vertical axis represents the effects of SNPs on cardiac tumor: (A) the result of five different MR analyses for the relationship between family Rikenellaceae id.967 (ebi‐a‐GCST90016950) and cardiac tumor; (B) the result of five different MR analyses for the relationship between genus Eubacterium brachy group id.11296 (ebi‐a‐GCST90016996) and cardiac tumor.

**FIGURE 3:**
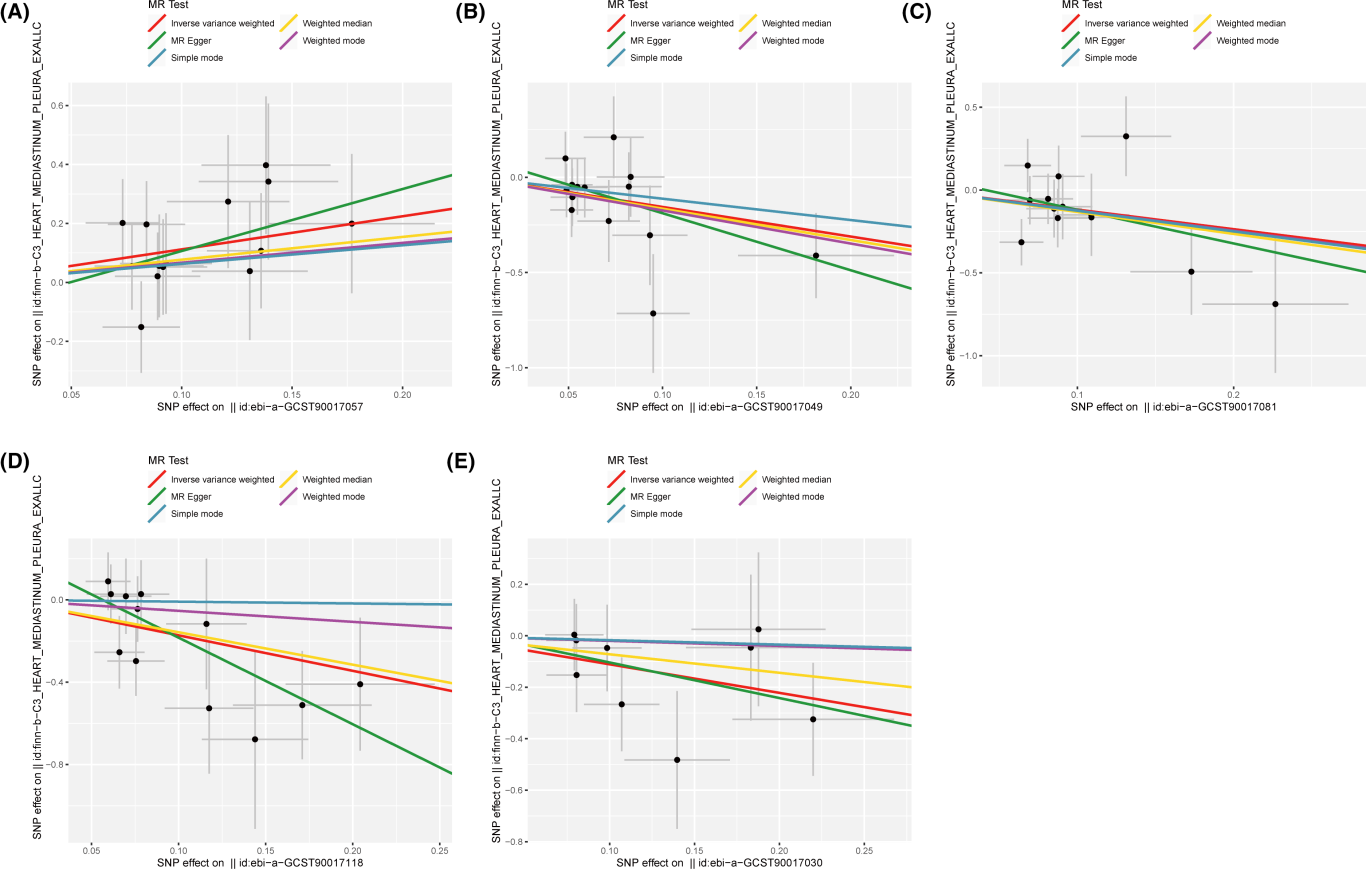
Association of gut microbiota with cardiac tumor in various Mendelian randomization (MR) analysis Models. Scatter plots represents the causal relationship between gut microbiota with cardiac tumor in Inverse variance weighted, weighted median, MR‐Egger, weighted mode and simple mode test. Horizontal ordinate represents the effects of SNPs on gut microbiota; Vertical coordinates represents the effects of SNPs on cardiac tumor: (A) genus Ruminococcaceae UCG009 id.11366 (ebi‐a‐GCST90017057); (B) genus Ruminiclostridium5 id.11355 (ebi‐a‐GCST90017049); (C) unknown genus id.1868 (ebi‐a‐GCST90017081); (D) phylum Verrucomicrobia id.3982 (ebi‐a‐GCST90017118); (E) genus Lactobacillus id.1837 (ebi‐a‐GCST90017030).

**FIGURE 4:**
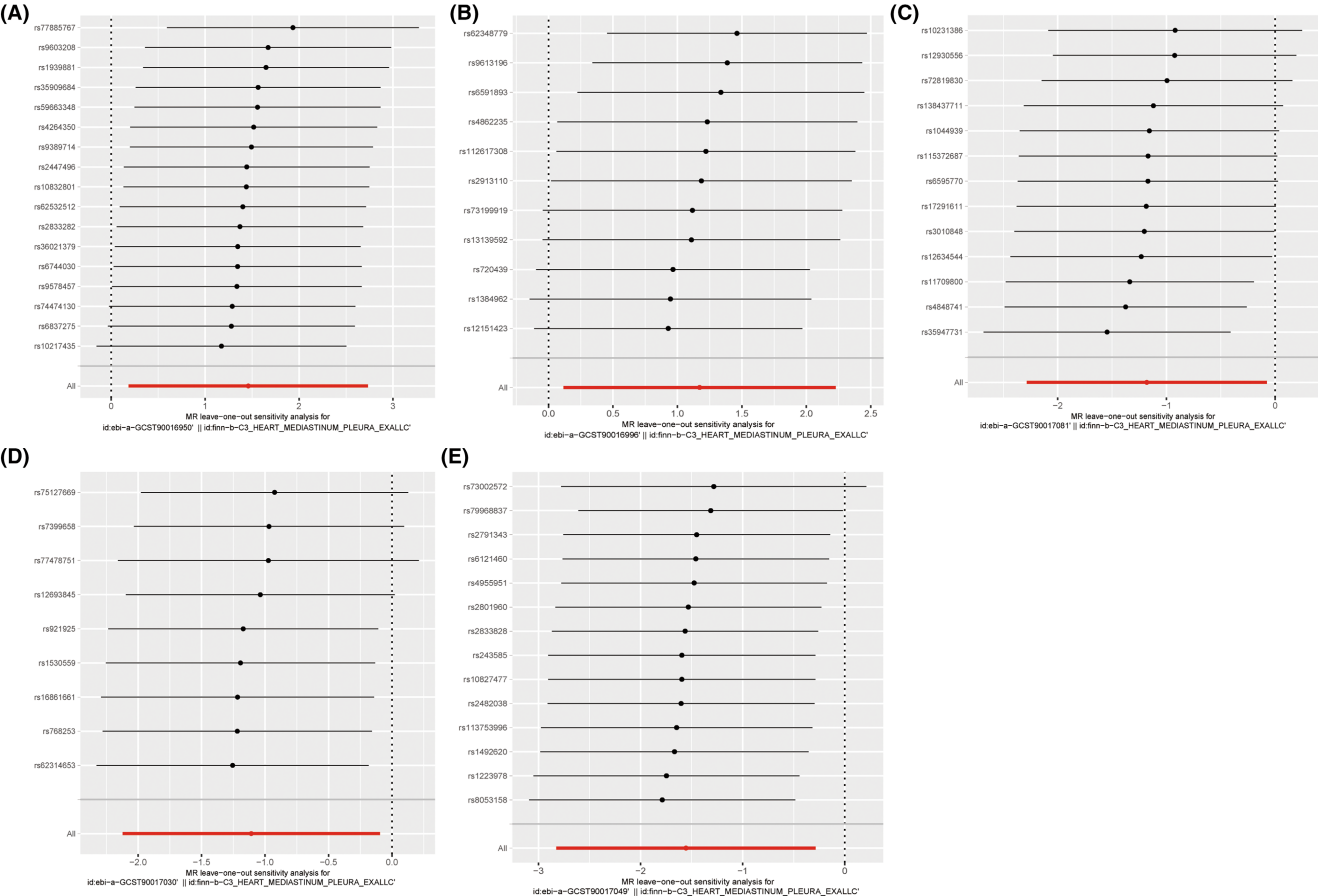
A leave‐one‐out sensitivity analysis for five different gut microbiota. Horizontal ordinate represents odds ratio between single SNP and cardiac tumor; vertical coordinates represents single SNPs corresponding to gut microbiota in genome‐wide association studies (GWAS) dataset. The datasets used for the analysis are as follows: (A) data set (ebi‐a‐GCST90016950): includes SNPs from family Rikenellaceae id.967; (B) data set (ebi‐a‐GCST90016996): includes SNPs from genus *Eubacterium* brachy group id.11296; (C) data set (ebi‐a‐GCST90017081): includes SNPs from unknown genus id.1868; (D) data set (ebi‐a‐GCST90017030): includes SNPs from genus *Lactobacillus* id.1837; (E) data set (ebi‐a‐GCST90017049): includes sNPs from genus *Ruminiclostridium*5 id.11355.

**FIGURE 5:**
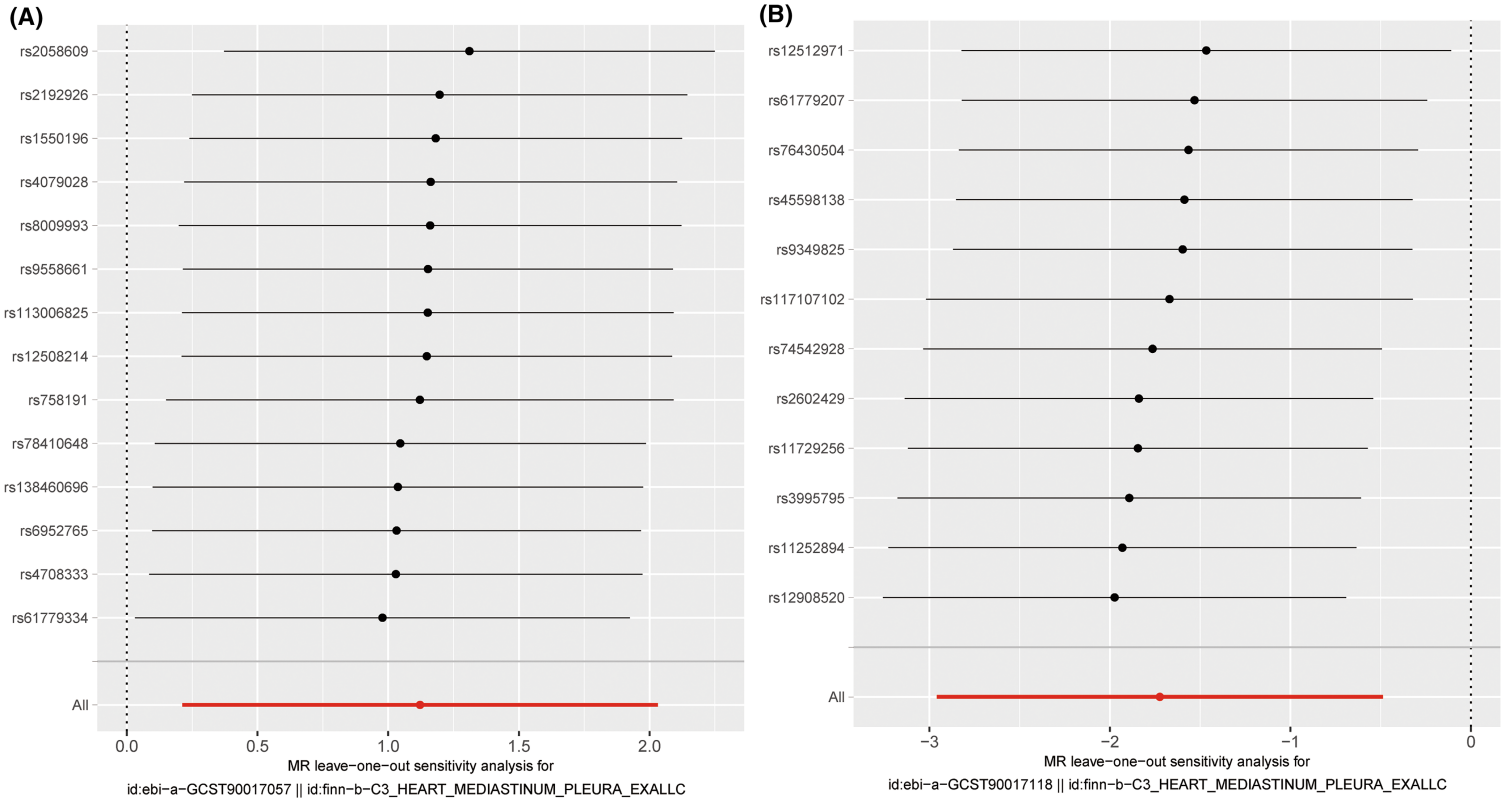
Single SNPs in two gut microbiota have little effect on cardiac tumor. Horizontal ordinate represents odds ratio between single SNP and cardiac tumor; vertical coordinates represents single snps corresponding to gut microbiota in genome‐wide association studies (GWAS) dataset. The datasets used for analysis are as follows: (A) Data set (ebi‐a‐GCST90017057): includes SNPs from genus *Ruminococcaceae* UCG009 id.11366; (B) data set (ebi‐a‐GCST90017118): includes SNPs from phylum Verrucomicrobia id.3982.

The scatter plot demonstrated consistent directions for all gut microbiota communities, except for genus *Eubacterium* brachy group id.11296 and family Rikenellaceae id.967 using different MR analysis (Figures 2 and 3). The MR‐Egger method, characterized by wider confidence intervals, was primarily employed to evaluate horizontal pleiotropy. The MR‐Egger intercept test and MR‐PRESSO global test indicated no horizontal pleiotropy of all results (*p* > 0.05) (Table 1). Similarly, all heterogeneity tests indicate *Q* values greater than 0.05, indicating the absence of heterogeneity among all the SNPs analyzed (Table 1). Finally, we performed the leave‐one‐out analysis to further estimate the stability of above results. It indicated that removing any single SNP in did not significantly affect the results of genus *Ruminococcaceae* UCG009 id.11366 and phylum Verrucomicrobia id.3982 (Figures 4 and 5). In conclusion, the genus *Ruminococcaceae* UCG009 increases the risk of cardiac tumors, while the phylum Verrucomicrobia reduces the risk of malignant cardiac tumors.

## 4 DISCUSSION

Malignant cardiac tumors are often overlooked in clinical diagnosis and treatment due to their low incidence. However, their outcomes and prognosis significantly impact human health and quality of life. Therefore, unraveling the molecular mechanisms underlying malignant cardiac tumors is crucial for better understanding their occurrence and progression. Dysregulation of gut microbiota has been identified as one of the key factors contributing to tumorigenesis.^12^, ^29^ A growing body of evidence indicates that the gut microbiota plays a crucial role in modulating the occurrence and progression of tumors, with its connection to metabolites becoming increasingly apparent. In a gastric cancer rat model induced by MNNG exposure, specific microbial species decreased significantly, while metabolites related to lipid metabolism and the PPAR signaling pathway exhibited notable changes.^30^ Yang et al. reported increased abundance of certain microbes positively correlated with amino acid and glucose metabolism but negatively correlated with fatty acid metabolism.^31^ Additionally, Peptostreptococcus anaerobius produces the tryptophan metabolite trans‐3‐indoleacrylic acid, which acts as an endogenous ligand for AHR, leading to the upregulation of ALDH1A3 and promoting colorectal carcinogenesis.^32^ Some studies have suggested the involvement of gut microbiota in tumor immunotherapy, affecting CD8^+^ cell activation and anti‐PD‐1 responses in non‐small cell lung cancer.^33^ Despite limited research on the role of gut microbiota in cardiac tumors, our study indicates a causal relationship between gut microbiota and the incidence of cardiac tumors. Notably, we found that the genus *Ruminococcaceae* increases the risk of malignant cardiac tumors, while the phylum Verrucomicrobia is associated with a reduced risk. These findings underscore the importance of regulating gut microbiota homeostasis in the diagnosis, prevention, and treatment of malignant cardiac tumors.

Furthermore, an increasing number of studies have revealed a possible relationship between the gut microbiota selected in our study and multiple diseases. For example, *Ruminococcaceae*, one of the earliest discovered bacteria in the stomach, plays a crucial role in metabolism.^34^, ^35^ It obtains nutrients by breaking down cellulose in the host's digestive system. Although *Ruminococcaceae* UCG‐009 has not been extensively studied in tumors, it plays a significant role in the regulation of blood glucose and lipids. In conditions of hyperlipidemia, the relative abundance of *Ruminococcaceae* UCG‐009 decreases after simvastatin treatment.^36^ In atherosclerosis, supplementation with the probiotic strain Lactobacillus can inhibit the abundance of *Ruminococcaceae* UCG‐009, reducing the levels of trimethylamine‐N‐oxide (TMAO), a gut metabolite associated with atherosclerosis risk.^37^, ^38^ In terms of blood glucose regulation, *Ruminococcaceae* UCG‐009 seems to have a blood glucose‐lowering effect. For example, treatment with Lycium barbarum polysaccharide (LBP) can induce an increase in the abundance of Ruminococcaceae UCG‐009 and reduce the abundance of HOMA‐IR, HDL‐C, ALT, AST, TC, and lipopolysaccharides (LPS).^39^ Moreover, Exposure to certain medications can also affect the relative abundance of *Ruminococcaceae* UCG‐009. For instance, nicotine significantly increases enrichment of *Ruminococcaceae* UCG‐009 in a high‐fat diet condition but not under normal chow diet feeding.^40^ Chlorpyrifos inhibits the relative abundance of *Ruminococcaceae* UCG‐009, leading to dysregulation of the hypothalamic–pituitary–adrenal axis, immune response, intestinal barrier function, and endocrine system affecting lipid levels.^41^ The evidence suggests a close association between *Ruminococcaceae* UCG‐009 and the occurrence and progression of cardiovascular diseases.

On the other hand, Verrucomicrobia is a gram‐negative bacterium that is widely present in the environment and is the third most abundant microbial group in the gut microbiota of healthy individuals.^42^, ^43^ Recent studies have reported the functional role of “Lentimonas” sp. CC4, a Verrucomicrobia strain including glycoside hydrolases, sulfatases, and carbohydrate esterases, in alginate degradation.^44^ In addition, Verrucomicrobia has been found to promote the expression of the regulatory T cell transcription factor Foxp3, suggesting its potential involvement in the immune system.^45^ However, research on this bacterial group's role in human diseases is limited, and to date, no studies have reported an association between Verrucomicrobia and tumors.

## 5 LIMITATIONS

In this study, we discovered that decreased the Verrucomicrobia microbiota and increased *Ruminococcaceae* UCG‐009 induce the occurrence of cardiac tumors. However, our investigation still has certain limitations. For instance, although we utilized the sole GWAS data available for cardiac tumors, the relatively low incidence rate of these tumors resulted in a limited number of cases in our study. Therefore, we were unable to conduct more detailed investigations into staging and subtypes, which could potentially impact the results to some extent, albeit unavoidably. To ensure the accuracy of this study, we conducted extensive sensitivity analysis and controlled for confounding factors. Although We have identified that aberrant gut microbiota led to malignant cardiac tumorigenesis, further investigation is imperative to unravel the underlying molecular mechanisms responsible for the observed phenomena.

## AUTHOR CONTRIBUTIONS

**Yongfei Song:** Conceptualization (lead); data curation (equal); formal analysis (equal); funding acquisition (equal); validation (equal); visualization (equal); writing – original draft (lead); writing – review and editing (equal). **Jiangfang Lian:** Funding acquisition (equal); investigation (equal); validation (equal); visualization (equal); writing – review and editing (equal). **Jiale Hu:** Data curation (equal); writing – review and editing (equal). **Chongrong Li:** Data curation (equal); writing – review and editing (equal).

## FUNDING INFORMATION

This study was supported by the National Natural Science Foundation of China [grant number 82300347], the Natural Science Foundation of Ningbo [Grant Number 2021J296], the Natural Science Foundation of Zhejiang Province [grant number LQQ20H160001] and the Medical Health Science and Technology Project of Zhejiang Provincial Health Commission [grant number 2021KY306] and Huadong medicine Joint Funds of Zhejiang Provincial Natural Science Foundation of China [grant number LHDMZ24H020001], Ningbo Key Laboratory of Molecular Target Screening and Application [grant numebr 2023‐BZDS], Science Foundation of Lihuili Hospital [grant number 2022ZD004].

## CONFLICT OF INTEREST STATEMENT

The authors declare no conflicts of interest.

## ETHICS STATEMENT

The present study relied solely on publicly accessible data that had already undergone ethical approval from a relevant ethics committee in previous participant studies. These studies followed the principles of ethical conduct in human investigation. As a result, no further ethical clearance was required for the current investigation, as the data used had been appropriately anonymized.

## Supporting information


Data S1.

Data S2.

## Data Availability

All data will be acquired from the corresponding author.
